# Usefulness of fetal magnetic resonance imaging for postnatal management of congenital lung cysts: prediction of probability for emergency surgery

**DOI:** 10.1186/s12887-018-1085-z

**Published:** 2018-03-08

**Authors:** Chiyoe Shirota, Takahisa Tainaka, Toshiki Nakane, Yujiro Tanaka, Akinari Hinoki, Wataru Sumida, Naruhiko Murase, Kazuo Oshima, Kosuke Chiba, Ryo Shirotsuki, Hiroo Uchida

**Affiliations:** 1 Department of Pediatric Surgery, Nagoya University Graduate School of Medicine, 65 Tsurumai-cho, Showa-ku, Nagoya, 466-8550 Japan; 2 Department of Radiology, Nagoya University Graduate School of Medicine, Nagoya, Japan

**Keywords:** Congenital lung cysts, Fetal MRI, Emergency lobectomy, CCAM

## Abstract

**Background:**

Emergency rescue lung resection is rarely performed to treat congenital lung cysts (CLCs) in neonates. Many reports have described fetal CLC treatment; however, prenatal predictors for postnatal respiratory failure have not been characterized. We hypothesized that fetal imaging findings are useful predictors of emergency surgery.

**Methods:**

We retrospectively studied patients with CLC who underwent lung surgery during the neonatal period in our hospital between January 2001 and December 2015. The demographic data, fetal imaging findings, and intra- and postoperative courses of patients who underwent emergency surgery (Em group) were compared with those of patients who received elective surgery, i.e., non-emergency surgery (Ne group).

**Results:**

The Em group and Ne group included 7 and 11 patients, respectively. No significant difference was noted in gestational age, time at prenatal diagnosis, birth weight, and body weight at surgery. The volumes of contralateral lung per thoracic volume were significantly smaller in the Em group than in the Ne group (*p* = 0.0188). Mediastinal compression was more common in the Em group (7/7) than in the Ne group (4/11) (*p* = 0.0128).

**Conclusions:**

This is the report describing neonatal emergency lobectomy in patients with CLC evaluated by fetal MRI using the lung volume ratio and mediastinal shift. In patients with CLC, mediastinal shift and significant decreases in contralateral lung volumes during the fetal stages are good prenatal predictors of postnatal emergency lung resection.

## Background

With the development of fetal ultrasonography, congenital lung cysts (CLCs) have been more frequently detected before birth. Fetal ultrasonography and magnetic resonance imaging (MRI) have been employed to determine prognosis after birth and whether interventions are required [1, 2]. CLCs are characterized by various patterns, ranging from patients without respiratory symptoms at birth to those with severe respiratory failure due to displacement of the contralateral lung [3].

Specific respiratory management and surgery for this rare disease in neonates are available only at advanced medical facilities, and elective Caesarean delivery is often required to prepare for sufficient medical intervention.

Predicting these patterns before birth could help determine treatment strategies and thus improve prognosis. Emergency rescue lung resection is rarely performed in neonates with CLCs. Many reports have described fetal treatment for CLC; however, prenatal predictors for postnatal respiratory failure have not been characterized [4–6]. We hypothesized that fetal imaging would help predict the requirement for emergency surgery.

Several reports described prenatal evaluation of CLC using ultrasonogram findings such as the lung thoracic ratio and cystic adenomatoid malformation volume ratio (CVR) [7–9]. Ultrasonography can be performed repeatedly with ease and is non-invasive; however, the accuracy of readings depends on the level of competency and technique of the practitioner. Fetal MRI is costly, and the time required to perform each scan limits its application; therefore, unlike ultrasonography, it cannot be performed repeatedly. However, fetal MRI exhibits several advantages over ultrasonography. Because the images obtained are almost three-dimensional, they are suitable for measuring volume and enable more accurate and objective evaluations. Although fetal MRI has been used for prenatal evaluation of the severity of congenital diaphragmatic hernia, its utility in evaluating the prognosis of CLC is unclear [10, 11]. The purpose of this study was to evaluate the severity and prognosis of CLC using fetal MRI, and thereby determine the appropriate treatment strategy.

## Methods

Prior to this study, all protocols were approved by the ethics review board at our institution. The medical records of patients who had undergone lobectomy for CLC treatment during the neonatal period in our department between January 1, 2001 and December 31, 2015 were retrospectively reviewed. In order to avoid thoracic deformity and early infection of CLC, we performed scheduled thoracoscopic surgery at near the end of neonatal period for patients who had a stable cardiorespiratory state. We performed emergency thoracoscopic surgery for patients who had an unstable respiratory state at early neonatal period or before scheduled surgery. We performed morphometric analyses of MRI images to assess volumes (ml) of the affected lesion, affected lung, contralateral lung, thoracic cavity, and mediastinal compression. We calculated the volume of the affected lung per thoracic cavity, volume of the affected lesion per thoracic cavity, and volume of the contralateral lung per thoracic cavity. The characteristics of patients (gestational age, time at prenatal diagnosis, birth weight, age at surgery, and body weight at surgery) and peri- and postoperative courses (surgery time, blood loss, duration of intubation [days], duration of respiratory support [days], and postoperative hospital stay) were compared between the patients who underwent emergency surgery for postnatal respiratory failure—the emergency surgery (Em) group—or those who underwent elective surgery—the non-emergency surgery (Ne) group. Analyses of the MRI images were performed by radiologists who were blinded to the patients’ background. The volumes were calculated using SYNAPSE VINCENT® FUJIFILM MEDICAL SOLUTION analytical software by easily combining values obtained from traced individual MRI images. A heart clearly displaced from the midline and mediastinal displacement diagnosed by a radiologist were defined as mediastinal shift. Statistical analysis was performed with the Wilcoxon test and Fisher exact test using JMP Pro 11, Statistical Discovery from SAS.

## Results

All fetal MRI was performed during the third trimester. Of 21 patients who underwent surgery within 30 days after birth, three patients without prenatal diagnosis were excluded. The Em group and the Ne group included 7 and 11 patients, respectively. No significant difference was noted in gestational age, time at prenatal diagnosis, and birth weight. The age at surgery was significantly younger and the body weight at surgery was significantly smaller in the Em group than in the Ne group. (Table 1) In the emergency surgery group, prenatal diagnoses were 4 cases of congenital pulmonary airway malformation (CPAM) type I, 2 cases of CPAM type II, and 1 case of CPAM type III; and pathological diagnoses were 4 cases of CPAM type I, 2 cases of CPAM type II, and 1 case of CPAM type III. In the elective surgery group, prenatal diagnoses were 4 cases of CPAM type I, 5 cases of CPAM type II, and 2 cases of CPAM type III; and pathological diagnoses were 4 cases of CPAM type I, 5 cases of CPAM type II, and 2 cases of CPAM type III.

**Table 1:** Characteristics of patients who underwent lobectomy within 30 days of birth

	Emergency operation group	Non-emergency operation group	*p value*
	*n* = 7	*n* = 11	
Gestational age at delivery (weeks)	38 (37–40)	39 (37–40)	0.0540
Time of prenatal diagnosis (weeks)	25 (21–32)	24 (20–34)	0.8553
Body weight at birth (g)	3028 (2484–3138)	3152 (2618–3483)	0.1473
Age at operation (days)	2 (0–27)	26 (24–30)	0.0005
Body weight at operation (g)	3028 (2484–3382)	4000 (3500–6405)	0.0006
	Median (range)		

The fetal images of both groups showed no significant differences in the volumes of affected lesions per thoracic cavity (Em: 0.174 and Ne: 0.274; *p* = 0.2090) and affected lung per thoracic cavity (Em: 0.228 and Ne: 0.108; *p* = 0.3927). The volumes of contralateral lung per thoracic cavity were significantly smaller in the Em group (0.166) than in the Ne group (0.195) (*p* = 0.0188). Mediastinal compression was more common in the Em group (7/7) than in the Ne group (4/11) (*p* = 0.0128). Concerning thoracoscopic lobectomy (Em; 4 cases, Ne; 11 cases), no significant intergroup differences were noted in surgery time (160 min and 206 min; *p* = 0.4687), although one thoracoscopic case in the Em group converted to thoracotomy due to deteriorating respiratory status. The volume of blood loss was significantly lower in the Ne group (2 ml) than in the Em group (18 ml) (*p* = 0.0360). The Em group required significantly longer intubation (19 vs 0 days; *p* = 0.0014), respiratory support (22 vs 0 days; *p* = 0.0023), and postoperative hospital stay (43 vs 10 days; *p* = 0.0085) than the Ne group (Table 2). The numbers of patients followed up for longer than 1 year after surgery were 4 and 11 in the Em group and the Ne group, respectively. No patients reported problems with daily living and growth.

**Table 2:** Comparison of fetal magnetic resonance imaging examinations and pre-operative and post-operative status between the emergency operation and non-emergency operation groups

	Emergency operation group	Non-emergency Operation group	*p* value
	*n* = 7	*n* = 11	
Volume of affected lesion/thoracic cavity (ml)	0.174(0.000–0.445)	0.274 (0.152–0.389)	0.2090
Volume of affected lung/thoracic cavity (ml)	0.228(0.013–0.554)	0.108 (0.008–0.337)	0.3927
Volume of contralateral lung/thoracic cavity (ml)	0.166 (0.051–0.199)	0.195 (0.139–0.267)	0.0188
Positive for mediastinal compression (n)	7	4	0.0128
Positive for hydrops (n)	2	0	0.1373
Blood loss (ml)	18 (1–114)	2 (0–41)	0.0360
Number of intubation days after operation	19 (1–22)	0 (0–3)	0.0014
Number of respiratory support days after operation	22 (6–80)	0 (0–39)	0.0023
Postoperative hospital stay (days)	43 (21–109)	10 (7–77)	0.0085
Underwent thoracotomy (n)	3	0	
Underwent thoracoscopic surgery (n)	4	11	
Operative time for thoracoscopy (min)	160 (75–296)	206 (83–441)	0.4687
	Median (range).		

In three patients who underwent fetal therapy, emergency surgery was performed at 1, 2, and 4 days of age. In the former two patients, a shunt tube was inserted at 25 and 29 weeks of gestation, and in the latter patient, cyst puncture was performed at 25 weeks of gestation. Fetal MRI was performed at 34, 34, and 39 weeks of gestation and revealed a mediastinal shift in all patients. The ratios of the contralateral lung volume to the thoracic cavity were 0.102, 0.147, and 0.207, respectively. The latter patient underwent MRI at 27 weeks of gestation, prior to fetal therapy, which indicated that the ratio of the contralateral lung volume to the thoracic cavity was 0.164, which improved to 0.207 with fetal therapy. Even though contralateral lung volume increased with fetal therapy, emergency lung resection was required.

One patient exhibited fetal hydrops on ultrasonography at 25 weeks of gestation with marked cardiac displacement, both of which improved by 33 weeks. The ratio of the contralateral lung volume to the thoracic cavity was 0.033 at 26 weeks and 0.190 at 33 weeks. Rupture of the membrane at 37 + 4 weeks resulted in vaginal delivery, with the neonate weighing 3120 g and in good respiratory condition. Although surgery had been scheduled, emergency surgery with a partial resection of the left superior and inferior lobes was performed because of respiratory difficulty on day 27 after birth.

Seven patients required emergency surgery at the age of 0, 0, 2, 2, 2, 4, and 27 days, four of whom required continuous one-lung ventilation until the surgery. The median ratio of the contralateral lung volume to the thoracic cavity was significantly lower in the Em group than in the Ne group. The respiratory status of six patients rapidly deteriorated soon after birth.

## Discussion

In fetal imaging, poor resolution can prevent diagnosis of CLC. Diagnosis using ultrasonography is further complicated by artifacts caused by amniotic fluid volume, the form of the maternal body, and fetal movement—issues that are not applicable to MRI. Furthermore, the contrast of a lung cyst (LC) and the affected lung is sometimes poor and indistinct, making it difficult to accurately measure the LC and the affected lung volume. In contrast, the normal lung is easily delineated. We compensated for the influence of lung volume fluctuations by measuring the thoracic volume and calculating the thoracic ratio. Thus, evaluation of the volume of the contralateral (not affected) lung and the thoracic volume provides accurate data.

The ultrasonic CVR can be repeatedly evaluated, and it is a convenient and very useful predictor. On the other hand, for congenital diaphragmatic hernia, various MRI techniques have been previously reported, of which lung volume was reported to be an effective predictor for prognosis [10]. In CLC, MRI can also be utilized to measure the congenital CVR, a useful indicator of the fetal treatment and ex-utero intrapartum treatment procedure [6, 9, 12]. CVR values over 1.6 or 2.0 have been reported to be high risk factors [6, 9, 12]. CVR can be calculated using the formula length × height × width × 0.52 / the head circumstance, using either MRI or echo images, and is a volume calculation based on the values obtained from cross sections, which differs from the three-dimensional volume measurement achieved using MRI. In our study, the ultrasonic CVR, which acts as the index of postnatal respiratory condition, was only measured in some cases. The results of our ultrasonographic examinations cannot be compared with the results of maternal MRI. Maternal MRI, which is not affected by the skill of the technician, is routinely performed in our institution when abnormalities are suspected in the fetus. To reduce the effect of the MRI performance time, the thoracic volume was used instead of the head circumstance.

Because we believed that surgery could be conducted efficiently if we waited about 30 days after birth, elective lobectomy was performed in the neonatal period (near 30 days) to avoid the risk of early cystic infection in this study period. In the neonatal period, the infant’s body is small, so the operation is slightly challenging to perform; however, as the surgery does not increase the risk of complications, we believe it is feasible. In our current strategy for the treatment of congenital lung cystic disease without respiratory distress, we perform delayed lobectomy within 3–6 months to make it easier for the surgeon to perform the operation when the infant’s body is a little bigger.

With advances in imaging analysis, volume measurement can be relatively easily applied, and based on the three-dimensional MRI images acquired, volume measurement could be widely used in the future. Future studies are expected to validate pulmonary volume as a predictor for the treatment and prognosis of CLC, and effective disease evaluations should be based on established reference values.

According to our results, CLC patients requiring emergency surgery for imminent respiratory failure after birth exhibited reduced fetal contralateral lung and mediastinal shift volumes. The Em group had longer postoperative hospital stays and required respiratory support for longer periods because of possible immaturity of the contralateral lung.

Patients in the Em group required one-lung ventilation or cyst puncture to maintain general condition sufficient for adequate surgery as early as possible. Such treatment is available only at limited medical facilities with an advanced system. Thus, if the requirement of emergency rescue lung surgery is prenatally determined, we cannot only select an appropriate birthing facility but also determine the type of delivery, such as elective Caesarean delivery, and timing of delivery based on the preparation of planned medical intervention.

Several studies have recommended early surgery even in asymptomatic cases based on the risk of pneumonia associated with non-treatment and reported no difference in surgery outcomes [13–19], whereas others recommended conservative management without surgery in asymptomatic cases [20] or waiting until 4–8 weeks after birth, reporting fewer complications when surgery was delayed by at least 3 months of birth [8, 21]. In the present study, we performed neonatal lobectomy in infants without respiratory distress; however, in the future, delaying lobectomy until 3–6 months of birth may not be associated with adverse outcomes.

In some cases of CLC diagnosed prenatally, we sometimes experience time-course change in cyst size detected by fetal ultrasonography. Although such possible time-course change in cyst size could affect the accuracy of our evaluation, a significant change in cyst size was not observed on a fetal ultrasonogram from the time of the third-trimester MRI scan to delivery. In our evaluation, we used MRI results performed in the third trimester even in the patients undergoing MRI examination multiple times; furthermore, instead of cyst size per se, the rate of the contralateral lung relative to the thoracic cavity was used; therefore, bias is not considered to be an issue.

## Conclusions

This is the report describing neonatal emergency lobectomy in patients with congenital lung cysts detected by fetal MRI using lung volume ratio and mediastinal shift. We aim to improve the accuracy and prognostic capacity of this procedure by accumulating further clinical data. In patients with CLCs, mediastinal shift and significant decreases in contralateral lung volumes during the fetal stages are good prenatal predictors of postnatal emergency lung resection.

## Abbreviations

CCAM: Congenital Cystic Adenomatoid Malformation
CLC: Congenital Lung Cyst
CVR: Cystic Adenomatoid Malformation Volume Ratio
Em Group: Emergency Surgery Group
MRI: Magnetic Resonance Imaging
Ne Group: Non-emergency Surgery Group

## References

[1] Epelman M, Kreiger PA, Servaes S, Victoria T, Hellinger JC (2010). Current imaging of prenatally diagnosed congenital lung lesions. Semin Ultrasound CT MR.

[2] Ruano R, da Silva MM, Salustiano EM, Kilby MD, Tannuri U, Zugaib M (2012). Percutaneous laser ablation under ultrasound guidance for fetal hyperechogenic microcystic lung lesions with hydrops: a single center cohort and a literature review. Prenat Diagn.

[3] Halloran LG, Silverberg SG, Salzberg AM (1972). Congenital cystic adenomatoid malformation of the lung. A surgical emergency. Arch Surg.

[4] Vrecenak JD, Howell LJ, Khalek N, Moldenhauer JS, Johnson MP, Coleman BG (2014). Outcomes of prenatally diagnosed lung lesions in multigestational pregnancies. Fetal Diagn Ther.

[5] Witlox RS, Lopriore E, Oepkes D (2011). Prenatal interventions for fetal lung lesions. Prenat Diagn.

[6] Cass DL, Olutoye OO, Cassady CI, Moise KJ, Johnson A, Papanna R (2011). Prenatal diagnosis and outcome of fetal lung masses. J Pediatr Surg.

[7] Usui N, Kamata S, Sawai T, Kamiyama M, Okuyama H, Kubota A (2004). Outcome predictors for infants with cystic lung disease. J Pediatr Surg.

[8] Khalek N, Johnson MP (2013). Management of prenatally diagnosed lung lesions. Semin Pediatr Surg.

[9] Crombleholme TM, Coleman B, Hedrick H, Liechty K, Howell L, Flake AW (2002). Cystic adenomatoid malformation volume ratio predicts outcome in prenatally diagnosed cystic adenomatoid malformation of the lung. J Pediatr Surg.

[10] Akinkuotu AC, Cruz SM, Abbas PI, Lee TC, Welty SE, Olutoye OO (2016). Risk-stratification of severity for infants with CDH: prenatal versus postnatal predictors of outcome. J Pediatr Surg.

[11] Hayakawa M, Seo T, Itakua A, Hayashi S, Miyauchi M, Sato Y (2007). The MRI findings of the right-sided fetal lung can be used to predict postnatal mortality and the requirement for extracorporeal membrane oxygenation in isolated left-sided congenital diaphragmatic hernia. Pediatr Res.

[12] Cass DL, Olutoye OO, Cassady CI, Zamora IJ, Ivey RT, Ayres NA (2013). EXIT-to-resection for fetuses with large lung masses and persistent mediastinal compression near birth. J Pediatr Surg.

[13] Tsai AY, Liechty KW, Hedrick HL, Bebbington M, Wilson RD, Johnson MP (2008). Outcomes after postnatal resection of prenatally diagnosed asymptomatic cystic lung lesions. J Pediatr Surg.

[14] Vu LT, Farmer DL, Nobuhara KK, Miniati D, Lee H (2008). Thoracoscopic versus open resection for congenital cystic adenomatoid malformations of the lung. J Pediatr Surg.

[15] Conforti A, Aloi I, Trucchi A, Morini F, Nahom A, Inserra A (2009). Asymptomatic congenital cystic adenomatoid malformation of the lung: is it time to operate?. J Thorac Cardiovasc Surg.

[16] Komori K, Kamagata S, Hirobe S, Toma M, Okumura K, Muto M (2009). Radionuclide imaging study of long-term pulmonary function after lobectomy in children with congenital cystic lung disease. J Pediatr Surg.

[17] Saeed A, Kazmierski M, Khan A, McShane D, Gomez A, Aslam A (2013). Congenital lung lesions: preoperative three-dimensional reconstructed CT scan as the definitive investigation and surgical management. Eur J Pediatr Surg.

[18] Shanmugam G, MacArthur K, Pollock JC (2005). Congenital lung malformations--antenatal and postnatal evaluation and management. Eur J Cardiothorac Surg.

[19] Choudhury SR, Chadha R, Mishra A, Kumar V, Singh V, Dubey NK (2007). Lung resections in children for congenital and acquired lesions. Pediatr Surg Int.

[20] Ng C, Stanwell J, Burge DM, Stanton MP (2014). Conservative management of antenatally diagnosed cystic lung malformations. Arch Dis Child.

[21] Khosa JK, Leong SL, Borzi PA (2004). Congenital cystic adenomatoid malformation of the lung: indications and timing of surgery. Pediatr Surg Int.

